# UK daily meteorology, air quality, and pollen measurements for 2016–2019, with estimates for missing data

**DOI:** 10.1038/s41597-022-01135-6

**Published:** 2022-02-09

**Authors:** Manuele Reani, Douglas Lowe, Ann Gledson, David Topping, Caroline Jay

**Affiliations:** 1 School of Management and Economics, The Chinese University of Hong Kong, Shenzhen, China; 2 Department of Computer Science, University of Manchester, Manchester, UK; 3 Research IT, University of Manchester, Manchester, UK; 4 Department of Earth and Environmental Sciences, University of Manchester, Manchester, UK

**Keywords:** Environmental monitoring, Atmospheric chemistry, Scientific data, Databases

## Abstract

In recent years, quantifying the impacts of detrimental air quality has become a global priority for researchers and policy makers. At present, the systems and methodologies supporting the collection and manipulation of this data are difficult to access. To support studies quantifying the interplay between common gaseous and particulate pollutants with meteorology and biological particles, this paper presents a comprehensive data-set containing daily air quality readings from the Automatic Urban and Rural Network, and pollen and weather data from Met Office monitoring stations, in the years 2016 to 2019 inclusive, for the United Kingdom. We describe (1) the sources from which the data were collected, (2) the methods used for the data cleaning process and (3) how issues related to missing values and sparse regional coverage were addressed. The resulting data-set is designed to be used ‘as is’ by those using air quality data for research; we also describe and provide open access to the methods used for curating the data to allow modification of or addition to the data-set.

## Abstract (summary)

**Table:** 

Measurement(s)	meteorology • air quality • pollen
Technology Type(s)	digital curation
Factor Type(s)	temporal interval
Sample Characteristic - Environment	air
Sample Characteristic - Location	United Kingdom

## Background & Summary

Understanding the ecological effects of air pollution requires the collection of a variety of air quality and meteorological measurements over considerable time periods and across wide geographical regions. This necessitates the complex preprocessing and integration of heterogeneous data from different sources into a single, accessible archive. Some of the challenges faced by data engineers during a data integration process include redundancy, inconsistency and missing values^1^. This *behind the scenes* aspect of data engineering, described as a ‘mundane labor’, is believed to occupy 50–80% of data scientists’ work^2^. Improving the data integration process, to allow research effort to be focused on knowledge discovery rather than the repeated creation of collection and cleaning methods, or even the repeated processing of the same data, is an ongoing Big Data challenge across many research domains^3–5^, and is a particular issue for air quality measurements^6^ and their interpolation^7^.

Sharing both the data itself and the related data processing methods is key to improving the efficiency and effectiveness of scientific discovery. This paper describes the process of curating a data-set that provides rapid access to air quality measurements drawn from the open Automatic Urban and Rural Network (AURN), integrated with other relevant variables that are pertinent to its interpretation. This data-set is already an important part of a number of ongoing projects, including those looking at the optimisation of sensor positioning, the honing of the level of detail required in pollutant speciation, and the relationship between hay fever symptoms and environmental variables (building on the work of Vigo *et al*.^8^). The data are likely to be directly relevant to a wide range of researchers working in this highly active area, and a principal aim of publishing the data is thus to improve the efficiency of research across the domain. By describing the process of collating and integrating the data, and providing the scripts created to perform this work, we also fulfil a secondary aim of improving the efficiency with which the research community can produce future data-sets.

‘Real-world’ data are perceived to be representative and reliable, but more frequently they are ‘partial and contingent’^9^. Air quality data is no exception, being both geographically sparse and frequently subject to equipment failures and timeouts. The cost of deploying and maintaining measurements of key pollutants can be significant, compromising the ease with which we can increase the density of measurements. As a result, discovering reliable trends and patterns in the effects of air quality is improved by collecting and prepossessing data over large geographical areas and over multiple years. Thus far, potentially due to its sporadic nature, few longitudinal research studies have been conducted in the UK using pollutant, pollen and weather data, and these have been limited to small regions such as a single city^10^. Consequently, UK-wide preprocessed and integrated data-sets are rare unless attached to a specific scientific study. The data-set presented here contains UK air quality, pollen and weather data collected from UK monitoring stations between January 2016 and December 2019, averaged for each day. We also report a methodology that can be used to increment the data-set in future years.

In the following section, we describe the construction of this integrated data-set in detail. We describe the data sources, how the data has been cleaned and processed, and how estimations have been made across all UK regions to mitigate sparse or missing data.

## Methods

Compiling the data-set involved the extraction of data from various sources, and managing missing data. Table 1 outlines the composition of the final data-set, the sources used, and temporal frequency and geographical coverage. In the rest of the section, we describe the data sources, extraction methods and the techniques used for dealing with missing data in further detail.

**Table 1:** Dataset Descriptions (for a description of *postcode regions* see the Estimating data across regions section).

dataset	variables	source	data frequency	postcode regions covered (of 126)
Pollen	(12 plants)	MIDAS	daily count	15
Pollutants	O_3_, NO_2_, SO_2_, PM_10_, PM_2.5_	DEFRA	22 cm daily mean/max	NO_2_: 75 Other: 52–57
Weather	temp, RH, pressure	MIDAS	22 cm daily mean/max	temp/RH: 98, pressure: 69

### Data sources and preparation

The pollen count and weather data-sets were taken from the Met Office’s MIDAS data-set (https://www.metoffice.gov.uk/), extracted via the University of Exeter MEDMI database (https://www.ecehh.org/research/medmi/ and https://www.data-mashup.org.uk). The source for the pollutant data-set was the UK Government’s air pollution monitoring Automatic Urban and Rural Network (AURN) (https://uk-air.defra.gov.uk/data/). Data was extracted from the AURN servers through a set of Python scripts that collect R data files for specific years and then apportion values into sites within regional authorities according to the metadata file held at the same location. Modelled pollutant data was also extracted from simulations made using the European Monitoring and Evaluation Programme for Transboundary Long-Range Transported Air Pollutants (EMEP) model (https://github.com/metno/emep-ctm). The model set-up and scripts for extracting all these data-sets are described in the Code Availability section (along with guidance on their usage).

The following sections describe these data-sets, detailing the various types of data within each one, the periods of data availability and the quantity of missing data. Missing values were due to data not being collected in an existing monitoring station for a particular period of time (e.g., on a particular day, the monitoring station was faulty, thus data were not collected for that day). In addition, during the data cleaning process, some data was deliberately removed and replaced with missing values; where this happens, and the reasons for this, are noted below. Although some data-sets that have been collated for this work are provided as hourly data, we have only included daily data for all data-sets in this work due to the size of the raw hourly data. Tools for replicating our workflows are provided for those who wish to access the hourly data. Missing values have been filled with imputed values for some data-sets; where this is the case it is also described in the relevant section. An overview of the regional coverage for each data-set is shown in Table 1 and a full csv file of regions and the number of sites for each, by measurement type, can be found in the published regional estimations data repository^11^.

#### Pollen count data-set

Pollen grain counts (in units of grains m^−3^ 24 hours^−1^) were available from 14 monitoring sites for 12 different pollen types: hazel (*Corylus* spp.), alder (*Alnus* spp.), willow (*Salix* spp.), birch (*Betula* spp.), ash (*Fraxinus* spp.), elm (*Ulmus* spp.), oak (*Quercus* spp.), plane (*Platanus* spp.), grass (Poaceae), nettle family (Urticaceae), mugwort (*Artemisia* spp.), and ragweed (*Ambrosia* spp.). These measurements were taken using volumetric Hirst-Burkard traps, using an airflow rate of 10 l m^−2^, and are estimated to have a collection efficiency between 60–90% (depending on the external wind speed)^12^. The data collection period for the pollen count monitoring stations is early March to early September in the years 2016 to 2019. It is not appropriate to use imputation to fill gaps in the pollen data-set both because of the sparsity of the monitoring stations, and because of the absence of measurements over the winter months (some genera, most notably hazel and alder can start their pollen seasons in January or February^13^). Consequentially, for the periods in which there are no measurements, and for missing values within the measurement periods (these constituted approximately 6% of the whole data-set), we have left the variables empty.

#### Pollutants data-set

We downloaded hourly measurement data for the pollutants O_3_, NO_2_, NOx (as NO_2_), SO_2_, PM_10_ and PM_2.5_. O_3_, NO_2_, NOx, and SO_2_ are all in units of *μ*g m^−3^, while PM_10_ and PM_2.5_ are in gravimetric units of *μ*g m^−3^. Gaseous components are provided to a precision of 0.00001 *μ*g m^−3^, particulates to a precision of 0.1 *μ*g m^−3^. The particulate measurements are taken with a variety of instruments at different sites, with correction factors applied to ensure equivalence between data-sets. We will not cover these here, but refer the reader to the source website (https://uk-air.defra.gov.uk/data/openair-data-definition). Data cleaning for these data-sets consisted of removing all negative or zero measurement values (with the assumption these are nonphysical). Such values are rare; the PM_2.5_ data had the most, at 1.3% of the total data count. We retained all stations which had the equivalent of at least 2 years of data for any pollutant (counted as the number of days that have at least one data point), giving us 74, 151, 151, 27, 69, and 72 measurement sites for the pollutants O_3_, NO_2_, NOx, SO_2_, PM_10_ and PM_2.5_ respectively (for these same pollutants, 9, 17, 17, 3, 23, and 14 stations respectively had less than 2 years of data and were therefore dropped). Missing values constituted 5.2%, 9.3%, 9.2%, 12.5%, 12.3% and 12.6% of the retained site data for pollutants O_3_, NO_2_, SO_2_, PM_10_ and PM_2.5_ respectively. Imputation methods were used to fill the missing values in the hourly data, following the methods described below, using AURN and EMEP data. From this hourly data, daily mean and maximum values were calculated for each station and pollutant. Daily data has been chosen here because the data-set is primarily designed for comparison with experience-sampled symptom data, which is rarely measured at a frequency greater than daily, and so using a higher temporal resolution would be inappropriate. It should be noted that the AURN network was designed to help local authorities understand how the air quality in their particular region is changing and evolving. The measurement sites have been chosen and located so that the measurements made will be representative of the air quality for the region (for the given environment of each site, e.g. urban roadside, suburban background, etc), but resource limitations have meant that this coverage is not comprehensive in the same way that the meteorological measurement network is.

#### EMEP modelled pollutants

Version v4.33 (201906) of the EMEP model, a 3-D Eulerian chemical transport model that integrates chemical processes with large-scale transport processes, was used for this study. This was driven by meteorological fields from the Weather Research and Forecasting (WRF) model (version 4.1.3), in turn driven by ERA-5 global meteorological data. The model setup consisted of two domains; a 50 km resolution domain covering all of Europe was used to create chemical boundary conditions for a second, higher resolution (3 km) domain covering the majority of the British Isles. Anthropogenic emissions for both domains were taken from the EMEP Centre on Emission Inventories and Projections (https://www.ceip.at/webdab-emission-database) emission database for 2017 (at a resolution of 0.1 × 0.1 degrees), which is provided with the EMEP model. For the second domain the UK National Atmospheric Emissions Inventory (NAEI; https://naei.beis.gov.uk/) for 2016 (at a resolution of 1 × 1 km) was used to replace the EMEP emissions for the UK, Atlantic, and North Sea regions. The four year simulation period was split into 18 two-month periods, each preceded by a 7-day spin-up period to initialise the chemical fields. We extracted hourly forecasts of the pollutants O_3_, NO_2_, SO_2_, PM_10_ and PM_2.5_ from the EMEP simulation data from the ground surface, at every listed AURN measurement station (272 stations). The hourly data has principally been generated for use in the imputation of the AURN measurement data (see the Imputation of Missing Values section below), however daily mean and maximum values calculated directly from this data are also included in the final dataset.

#### Meteorological data-set

We collected hourly data for the following measurements: air temperature (°C), dewpoint temperature (°C), and station pressure (hPa). All these measurements are stored in the MEDMI database to a precision of 0.1 °C or hPa. This data was extracted for all stations which have air temperature measurements; this leads to a small loss of pressure data (<1% of the total data-set), but enables better cleaning of the data-set. The MEDMI data-set has been built from a number of measurement networks, increasing spatial coverage, but leading to some duplication of data, where measurements for different networks are made at the same site, or the inclusion of data which has been sampled in a manner which is incompatible with our requirements.

The raw data-set has 11.44 million temperature measurements, 99% of which include dewpoint temperature, and 46% of which include pressure measurements. The two main measurement networks included in this data-set are the METAR and SYNOP networks (M. Sunter and C. Sarran, Met Office, personal communication, July 2020). Both include temperature and dewpoint temperature measurements, but (generally) only the METAR network includes pressure measurements too. Within the raw data-set there are 521,109 duplicated readings; generally these occur where METAR and SYNOP network instruments are co-located. The METAR network provides greater information, and generally has higher accuracy temperature measurements, so where there are duplicates we chose to retain the measurements with an associated pressure reading. For the 2 readings where both have pressure data, and the 8,567 where neither have pressure data, we simply keep the first reading, and drop the second.

There are 637,195 days of temperature data across the whole data-set (for all sites). 67% of these have 24 measurements per day, 28% have a single measurement per day, and 5% have between 2–23 hourly measurements. The vast majority of single daily temperature measurements are from a network of synoptic spot readings, taken daily at selected sites. Although we use imputation to create hourly data for other sites which are missing a lot of data, in this case we decided that these sites would not have enough information about diurnal variability of the measurements to do this sensibly, and so have removed the 166 measurement sites which have only these single daily temperature measurements.

Aside from removing duplicated readings and spot measurements, we also apply data filtering based on general data investigation. For this period and data-set two of these filters have been applied; when using our processing scripts to process data for different periods or data-sets we recommend users carry out their own data investigation to add to, or remove, the filters that will be described here.

The first filter is for site 117. This measurement station is in the Cairngorm mountains, and the measurements contain the most extreme relative humidity data (ranging from negative values through to RH > 110%) of the whole data-set. Rather than filter these suspect measurement points individually we have wholesale removed this site from the data-set (as population density is low in this region, so reducing the measurement coverage here is acceptable for our requirements). The second filter we apply is to remove all measurements which have temperature data lower than −20 °C. There are only 11 measurement points which are below this temperature, for site 18920 in March 2016. These are quite different to the rest of the data for that site, and so are likely to be measurement errors.

The whole data cleaning process removes 6.4% and 6.3% of the original temperature and dewpoint temperature measurements, respectively. Two pressure measurement datapoints are removed (this is the data count removed, not a percentage of the original data).

Following this cleaning, and adopting the same requirements as for the pollution data, we retained all stations which had the equivalent of at least 2 years of data for any of the meteorological variables (counted as the number of days which have at least one data point), giving us 319 measurement sites for temperature and dewpoint temperature, and 151 sites for pressure. The percentage of missing data at these selected sites is 4.9% and 1.6% for temperature and pressure respectively. The percentage of data points missing either temperature or dew point temperature data (both are required for calculating relative humidity) is 5.4%.

Sites which had at least 3.5 years’ data for all meteorological variables were selected to be reference sites for the imputation methods described below. This gave us 146 meteorological reference sites. Relative humidity was calculated from the air and dewpoint temperature data-sets, and daily mean and maximum values for air temperature, relative humidity, and station pressure were calculated for each station.

Relative humidity was calculated using the ratio of the vapour and saturation vapour pressures, which are calculated from the air and dewpoint temperatures respectively^14^. This calculation is carried out using the metpy python package. These calculated RH values compare reasonable well with the RH values provided in the Met Office data-set. The vast majority (>99%) were within 3% of the provided values, with a slight positive skew in the calculated values. The majority of the differences observed are likely to be due to using lower accuracy temperature data, which is rounded to 2 decimal places in the MEDMI database, for this calculation than that originally used by the Met Office.

### Imputation of missing values

Where a station did not capture data for a particular period of time, we have used imputation methods to fill the gaps in those measurements, using the python-based scikit-learn toolkit^15^. The criteria used to determine where imputation was appropriate are based on the type of measurement data at a station (i.e. the pollen measurements are not suitable for imputation due to the co-incident time periods of data unavailability, and relative humidity data is not imputed due to the shape of the distribution of values) and on a minimum data availability criteria, which we set to an equivalent of 2 years of measurements for that variable at that station. For each measurement data-set we have identified reference stations that have at least 3.5 years of measurements for that particular variable data-set.

For each station data-set that requires imputation we locate the 5 nearest reference stations for that data-set, and use these to impute the missing data for that station. For meteorological data-sets this is done separately for each data-set. For the pollutant data the other pollutant data for the station of interest and EMEP modelled pollutant data for that station are also included. The missing values in all linked data-sets are then imputed iteratively, until a solution is converged on for all of them.

The estimator used in the imputation process is Bayesian Ridge^16^. This uses a Gaussian prior for the coefficient weights, which allows for a slightly more stable estimator, but it can distort data-sets that do not initially have a normal distribution. To address this issue a transformer is used first, to create a normal distribution in the data-set (and the inverse used afterwards, to restore the original distribution). A quantile transformer, with “normal” output, is used for most of the data-sets, because the air and dew point temperature data (and ozone data in polluted locations) have bi-modal distributions which are not conducive to the use of a power transformer. A power transformer is used for the pressure data, however, because the quantile transformers clip outlier datapoints too harshly for this dataset (see https://scikit-learn.org/stable/modules/preprocessing.html#non-linear-transformation for more information on the different transformation methods that are available).

This process requires that there is suitable data at the reference sites for the time periods over which the imputed data is required. It also requires that there are strong correlations between the time series at the reference sites and the site being imputed. Because of this we have chosen not to use imputation to fill the gaps in the pollen timeseries, as the consistent lack of data for all sites across the winter months makes this process impossible. Validation of the imputation process was carried out by removing data for selected reference sites, and comparing the imputed values with the original data (see Technical Validation section). This validation process indicated that the imputation of SO_2_ data was generally not very reliable (Spearman’s correlation coefficient of <0.3). This is probably because of the sparsity of the SO_2_ measurements sites, and differing sources of this pollutant compared with the other pollutants. As such, we have chosen not to use imputation for gap-filling the SO_2_ data, although the option to do so is provided in our tools. The validation process also demonstrates the value of including EMEP data in this imputation process. Although there can be considerable absolute differences between the EMEP and measured pollutant data at the same site, the overall correlation between these is usually good, and this is what matters for the imputation process.

### Estimating data across regions

It is not possible for sensors recording all indicators to exist in every part of the UK, but environmental data research (such as population impact studies) often requires an estimation of the sensor values for specific target locations not necessarily close to a single sensor. In such cases (as opposed to cases caused by station failures: see the Imputation of Missing Values section for such cases) we propose a baseline estimation method that collects data from adjacent areas and uses these to infer the value in the target location.

To define *regions* in our estimation datasets, we adopted a standard UK regional subdivision which consists of 121 postcode areas (Table 2 in https://geoportal.statistics.gov.uk/documents/ons::ons-postcode-directory-may-2021-user-guide). Postcodes are used in the UK to direct mail and the first one or two alphabetical character(s) indicates the larger postcode areas. For instance, the postcode M13 9PL locates a specific address in the UK and the first letter (M) represents the Manchester area. These regions are not all equal in geographical extent and can have quite irregular shapes because their design has been influenced by both geography and population distributions within the UK. Although these irregularities can be a challenge for interpolating site measurements, due to their relationship with population rather than purely geographical features, they are still a potentially useful means for creating regional maps of environmental data when carrying out population impact studies.

**Table 2:** Sensor daily mean ranges for original and imputed measurements.

	original				inc. imputed values			
	mean	min	75%	max	mean	min	75%	max
NO_2_	24.65	0.12	33.67	201.00	24.65	0.04	33.72	201.00
NOx (as NO_2_)	50.90	0.15	63.53	1135.16	50.71	0.35	63.98	1124.91
O_3_	47.83	0.40	61.18	147.20	47.86	0.63	61.14	147.20
PM_10_	16.11	0.20	19.71	210.02	16.21	0.81	19.79	190.62
PM_2.5_	10.00	0.10	11.97	121.33	9.97	0.45	11.90	121.33
SO_2_	2.01	0.07	2.30	45.62	1.93	0.18	2.18	45.62
rel. hum.	82.95	24.40	89.76	106.77	83.06	24.40	89.81	100.00
pressure	1003.31	933.39	1014.15	1043.64	1003.50	933.39	1014.29	1043.64
temp.	9.93	−13.55	13.84	33.00	9.90	−13.55	13.83	29.04
*Alnus* spp.	1.66	0.00	0.00	1540.00	—	—	—	—
*Ambrosia* spp.	0.08	0.00	0.00	44.00	—	—	—	—
*Artemisia* spp.	0.32	0.00	0.00	145.00	—	—	—	—
*Betula* spp.	14.31	0.00	1.00	2644.00	—	—	—	—
*Corylus* spp.	0.77	0.00	0.00	303.00	—	—	—	—
*Fraxinus* spp.	3.77	0.00	0.00	874.00	—	—	—	—
*Platanus* spp.	2.09	0.00	0.00	911.00	—	—	—	—
Poaceae	22.57	0.00	16.00	991.00	—	—	—	—
*Quercus* spp.	5.57	0.00	1.00	713.00	—	—	—	—
*Salix* spp.	1.49	0.00	0.00	255.00	—	—	—	—
*Ulmus* spp.	0.54	0.00	0.00	198.00	—	—	—	—
Urticaceae	23.45	0.00	25.00	965.00	—	—	—	—

In this data-set we present both the site data at a single geographical point, and postcode area averaged data calculated using a baseline method that we called the *concentric regions method*. The *concentric regions* method works by imputing a regional value, using sensor values collected either from within that region or from its surrounding regions. Specifically, in cases where one wishes to infer the value of a particular indicator (e.g. PM_2.5_) in a postcode region that has one or more monitoring stations and therefore multiple values for that target location, the mean of those sensor values is used. In cases where a region does not contain any relevant monitoring stations, we infer the value for the target location by collecting the measurements from the postcode areas closest to the target region found to have one or more monitoring stations, again computing the mean of those measurements.

**Fig. 1:**
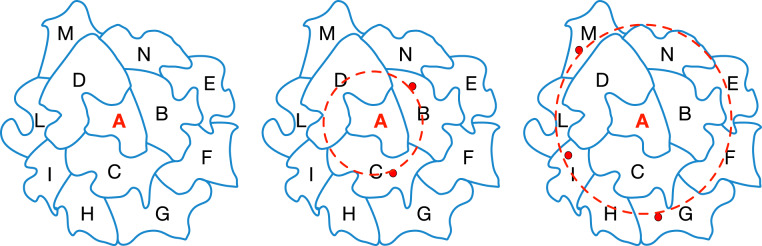
The concentric regions method illustrated on fictional postcode areas.

For example, Fig. 1 shows a fictional map of postcode areas. Given a postcode area *A*, a ring is defined as the list of postcode areas that have a physical boundary (adjacent) with *A*. Imagine we wish to know the PM_2.5_ value for postcode area A (Fig. 1 – left), but it does not have a monitoring station for this indicator. If we move out by one ring, to the adjacent postcode areas B, C and D (Fig. 1 – centre), we find 2 monitoring stations (red dots), one in area B and one in area C. In this case we calculate the average value of these stations to approximate a value for area A (i.e., the target location). Where there are no relevant monitoring stations in the immediately adjacent postcode areas, we move out by a further ring and look at the postcode areas adjacent to B, C and D. In this example these are E, F, G, H, I, L, M and N (Fig. 1 – right). Areas G, I and M have a monitoring station, so we take the average of these to approximate a value for A. In the first example we only need to move out by one ring to infer the value of A (rings = 1). In the second example we have to move out by two rings (rings = 2). If no sensors are found in this second ring, the process continues, using wider rings until either stations are found or no further outer regions exist.

No maximum is set for the number of rings as, firstly, there is no universal rational for what this upper bound should be, as it is likely to vary across different regions. Secondly, we report the number of rings used in the estimations dataset, allowing the user to set this limit, based on their own requirements. Thus an estimate, together with the confidence in the precision of such an estimate (represented by the rings) is provided. The numbers of rings required for each measurement are summarised in the Validation of Regional Estimations Method section.

In addition, as air quality measurement sites are divided into several different types (e.g. *urban traffic* or *rural background*), we provide two extra regional estimation air quality datasets, as described in the Data Records section (Regional estimates subsection), in which the datasets are divided into site types and regional estimations calculated separately. We implemented the above regional estimations baseline algorithm using Python and GeoPandas and describe how to access this code in the Code Availability section.

## Data Records

The data is published in two parts: firstly the AURN and Met Office cleaned and imputed diurnal data and secondly the daily estimates for each postcode area region, the former data-set being used to calculate the latter. These are described below.

### AURN and Met Office data

Cleaned and imputed versions of the AURN air quality and the Met Office pollen and meteorological data are available on Zenodo^17^. The dataset is provided in files: *turing_aq_daily_met_pollen_pollution_original_data.csv*, containing only original data; and *turing_aq_daily_met_pollen_pollution_with_imputation_data.csv*, containing original plus imputed data.

The dataset is presented in CSV format and the columns intended to be used as indexes are:

**time_stamp**:the date of the measurements on that row

**site_id**:a measurement site ID, corresponding to sites in the three networks: AURN (indicated by [AQ]), Met Office (indicated by [WEATHER]) or pollen (indicated by [POLLEN]).

The data columns are:

**air quality** (O3, PM10, PM2.5, NO2, NOXasNO2, SO2)

- daily mean and maximum values in *μ*g m^−3^ (all with “_max”, “_mean”, and “_flag” tags)
- AURN measurement data

**EMEP** (O3_EMEP, NO2_EMEP, SO2_EMEP, NOXasNO2_EMEP, PM2.5_EMEP, PM10_EMEP)

- daily mean and maximum values in *μ*g m^−3^ (all with “_max”, and “_mean” tags)
- EMEP model forecasts

**Pollen counts** (alnus, ambrosia, artemisia, betula, corylus, fraxinus, platanus, poaceae, quercus, salix, ulmus, urtica)

- daily pollen grain counts
- note that ‘urtica’ variable is for the Urticaceae pollen type
  **meteorological** (temperature, relative_humidity, pressure)
- daily mean and maximum values in °C, %, and hPa (all with “_max”, “_mean”, and “_flag” tags)
- Met Office measurement data

In the AURN and Met Office datasets, the “_flag” columns indicate the fraction of the hourly data points used to calculate the daily data points which have been imputed from hourly data. 0 = no imputed data; 1 = fully imputed data.

Site metadata is provided in the file *site_location_data.csv*. This data describes each sensor. This includes location (longitude and latitude as floats), site name (string used as sensor/site identifier), address, environment type and altitude. Each row represents a single sensor.

### Regional estimates

The regional estimated data-sets are also presented in CSV format and are available on Zenodo^11^, in six parts. The first and second parts comprise regional estimates based on cleaned AURN and Met Office data and regional estimates based upon cleaned and imputed AURN and Met Office datasets for all measurements, using all site types. The third and fourth parts are as above but only include air quality estimates, where the sites are split into four types: *industrial* which comprises *urban industrial* (9 sites) and *suburban industrial* (2 sites), *urban background* (48 sites), *rural background* (14 sites) and *urban traffic* (47 sites). The fifth and sixth parts are the region metadata file and the regional counts file.

**Region estimates (2 parts)**: The estimated values for each region, calculated using the concentric regions method described in the Estimating data across regions section. Each row represents a timestamp (string) and region id (string) combination. Each region id can be linked to a single region id in the *region metadata* file described above. The columns with measurement values as headers (e.g. PM2.5_mean or alnus) contain the estimated values (floats) for that measurement. Those columns prefixed with ‘rings_’ (e.g. rings_PM2.5_mean or rings_alnus) represent the number of rings used in the concentric regions method, when estimating the respective measurement. Estimates based only on original data are in the file *turing_regional_estimates_aq_daily_met_pollen_pollution_original_data.csv*; estimates based on original plus imputed data are in the file *turing_regional_estimates_aq_daily_met_pollen_pollution_imputed_data.csv*.

For the air quality estimation files (2 parts) that are stratified by site type (described above), the site types are included in the column header using the abbreviations: *industrial: Ind*, *urban background: UB*, *rural background: RB* and *urban traffic: UT*. For example: *NO2_Ind_mean*. Estimates based only on original data are in the file *turing_regional_estimates_aq_loc_type_daily_original_data.csv*; estimates based on original plus imputed data are in the file *turing_regional_estimates_aq_loc_type_daily_imputed_data.csv*.

**Region metadata**: The data describing each region (postcode area, e.g. ‘AB’). This includes the region identifier (string), geographical location/area (multi-polygon), a brief description of the postcode area, population, the set of nearest postcode areas and the country that the postcode is in. (These postcode data were extracted from a publicly available sources:

- https://www.doogal.co.uk/UKPostcodes.php
- https://www.freemaptools.com/download-uk-postcode-outcode-boundaries.htm
- https://www.gov.gg/population
- https://www.gov.je/Government/JerseyInFigures/Population/Pages/Population.aspx
- https://www.gov.im/media/1369690/isle-of-man-in-numbers-july-2020.pdf

Each row represents a single region. These data are in the file *postcode_district_data.csv*.

**Regional site counts**: The data describing each region (postcode area, e.g. ‘AB’) and the number of sites for each measurement, contained in each. These data are in the file *regional_site_counts.csv*.

## Technical Validation

Unit testing has been added to both the data download and processing code, and to the regional estimations code^18,19^. These have been automated using Github Actions (https://docs.github.com/en/actions), so that they are run each time the Github software repositories are updated. General testing of operational assertions, file paths, module availability, etc., have been added. Where unit tests have been applied for a specific method it is noted below.

### Measurement value ranges

To provide context for the validation of the data download cleaning and imputation methods, we firstly present a summary of the sensor value ranges (original and where imputed values are included), shown in Table 2. We show the mean, minimum, 75th percentile and maximum for each measure to demarcate the furthest outliers. Columns two to five show original (non-imputed) data and the final four columns describe data that include imputed values. All of the values are within the expected ranges and distribution patterns for each measurement type. Minor changes can be seen in the pollution and meteorological datasets, after imputation, with the range of values shrinking for all but one (NO_2_) of the measurements.

### Validation of imputation methods

The imputation methods were tested by removing a fraction of the data from the timeseries for specified reference sites, and statistically analysing the fit of imputed results with the original data. Data selection can be made randomly, or as a single contiguous block either at the start, end, or middle, of the timeseries. These tests have been wide-ranging, but are not exhaustive. They have been used to guide the choice of methods used and datasets imputed, but due to the heterogeneous nature of the measurement sites across the UK, and the nature of the data-sets being imputed, we cannot rule out the possibility that there will be some stations where the imputed data is a poor substitution for the measurement data.

Spearman’s Rank correlation coefficients have been calculated between the original and imputed datasets, for both the imputed hourly data, and for the daily mean and maximum data calculated from these (which can, depending on the method of data removal used, still contain some original data in the imputed values, as would happen for replacing missing data in real datasets).

**Fig. 2:**
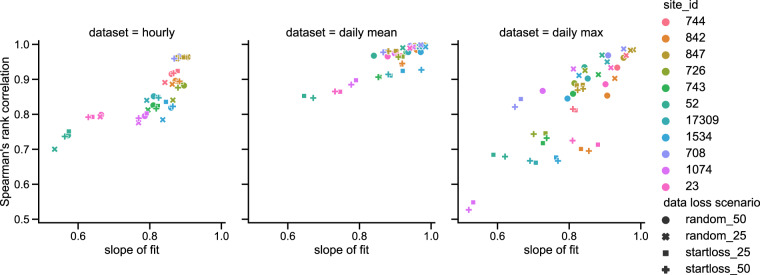
Spearman’s Rank correlation coefficient and slope of fit between relative humidity data calculated from original and imputed data. Point colours indicate site, and the shape indicates the data removal scenario.

From the meteorological datasets the relative humidity dataset, calculated from temperature and dew point temperature (both of which undergo the same data removal and imputation in these tests), is the most difficult to reproduce. We performed six different data loss scenarios (a combination of either 25 or 50% data removal, either randomly, at the start, or at the end of the time-series), for 14 reference sites. In all scenarios the temperature and pressure data had correlation coefficients greater than 0.9, and slopes between 0.9 and 1.0 (not shown here). For relative humidity data (shown in Fig. 2) the correlation coefficient is between 0.7–1.0 for the hourly data, improving to between 0.8–1.0 for the daily mean data, but worsening to between 0.5–1.0 for the daily maximum data. Slopes of fit generally remain between 0.5–1.0, with most between 0.8–1.0. For the hourly data the variation in fit seems to be most related to the site being imputed — it is perhaps how similar the site conditions are to those of its neighbours which determines how well reproduced the raw hourly data is. The fit of the daily mean data improves as some of the variability in the data is smoothed, but also as the original data is included in some of the calculations, so the datasets that have undergone random dataloss are generally a better fit. This effect is most pronounced in the daily maximum data, where the random and contiguous data loss scenarios almost form two separate clusters.

**Fig. 3:**
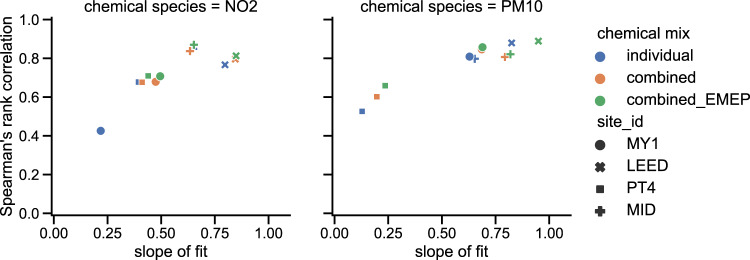
Spearman’s Rank correlation coefficient and slope of fit between hourly NO_2_ and PM_10_ data calculated from original and imputed data. Point colours indicate chemical mix used for imputation process, and the shape indicates measurement site. Data removal scenario was 50% from the start of the time period.

The pollutant datasets are generally more difficult to impute, due to the more spatially heterogeneous and complex sources and processes that these are subject to. Imputation of ozone, which has widespread free tropospheric sources, as well as local sources and sinks, is reasonably accurate. In tests, the Spearman’s Rank correlation coefficients, and slopes of fit, for the ozone hourly data are generally between 0.7–0.9, and 0.6–1.0, respectively (not shown). For most other pollutants, however, the ranges of these statistical measures for the hourly data are between 0.4–0.9 and 0.2–0.9, respectively. Imputing all chemical species together led to better correlations than imputing them individually, as did, to a lesser degree, including the EMEP data (see Fig. 3 for an example of this, for the scenario of 50% data removal from the start of the time series). The accuracy of the imputated values for SO_2_ is poor, with correlation coefficients as low as 0.2–0.7, and slopes of fit around 0.0–0.2 (not shown). This could be due to the sparsity of the SO_2_ measurement sites, so that correlations were being attempted between sites with very different environments. Imputing SO_2_ in concert with other pollutants and EMEP data does improve the fit, but not to the degree that it performed similarly to the other pollutant datasets. Because of this, the imputed SO_2_ data has not been included in this dataset.

Calculations of daily mean and maximum values for the pollutant datasets depend as greatly on the style of data removal as for the meteorological datasets. For random data removal, even at 50%, the correlations for these are in the range of 0.8–1.0, with slopes of fit between 0.6–1.0. For contiguous data removal scenarios the fits were much more similar to those for the pollutant hourly data, with SO_2_ standing out as the least correlated.

### Validation of regional estimations method

The region estimated data is tested using two methods: automated unit testing, to ensure the software is functioning as expected; and higher level statistical analysis examining the number of rings required and differences between known and estimated sensor measurement values, to check confidence levels and the validity of the estimation method.

Unit testing is used to check that our approach to finding areas and their adjacent regions is accurate. A set of edge case region types were created, including overlapping regions, non-touching regions and islands (e.g. Isle of Man). In addition, a set of random ordinary regions were selected. For each of these test regions, the expected estimation results (or errors) are also stored. The test scripts process this array of test files and check whether the estimation results match those expected. The full set of test regions, scripts and expected results can be viewed in the ‘test’ section of the region_estimators repository^19^.

To indicate whether the regional estimation method used to produce the data functioned as intended, some basic descriptive statistics are presented. These are provided for a representative subset of the datasets, selected as follows. For pollutants, a *gaseous* pollutant (NO_2_) and both *particulate* measures (PM_10_ and PM_2.5_) are selected as these two types have slightly different sources and concentric regions behaviours, so we can observe how well each are simulated. For the meteorological datasets we selected temperature and pressure, as the former is subject to local variation and is therefore more difficult to estimate using our simple *concentric regions* approach and the latter varies on a larger scale, making them easier to estimate. The two pollen species (*Alnus* spp. and Urticaeae) are selected randomly, as the many factors which will have an impact on this method (such as source distribution, seasonal variability, atmospheric transport processes, etc) are expected to have similar influences for all pollen types.

As outlined in the Estimating data across regions section, the number of concentric regions (or *rings*) used to calculate estimations can be used to represent an approximate confidence level, with lower numbers of rings generally indicating a higher level of confidence. Tables 3, 4 show the mean, minimum, maximum and standard deviations of the number of rings used, for each of the selected measurements, using the original and imputed datasets. Table 3 is for all seven selected measurements, using all site types uniformly. Table 4 shows only the three pollutant measurements, using the regional estimates datasets split by site type (as described in the Estimating data across regions section).

**Table 3:** Number of rings required for *concentric regions* estimations.

measur.	original				inc. imputed values			
	mean	std	min	max	mean	std	min	max
NO_2_	0.39	0.49	0	2	0.33	0.47	0	1
PM_10_	0.71	0.61	0	5	0.69	0.60	0	2
PM_2.5_	0.63	0.57	0	5	0.59	0.55	0	2
temp.	0.23	0.44	0	2	0.19	0.41	0	2
pressure	0.49	0.60	0	3	0.47	0.60	0	2
*Alnus* spp.	1.75	1.44	0	16	—	—	—	—
Urticaceae	1.75	1.44	0	16	—	—	—	—

**Table 4:** Number of rings required for *concentric regions* estimations.

type	measur.	original				inc. imputed values			
		mean	std	min	max	mean	std	min	max
Ind	NO_2_	1.88	1.27	0	8	1.86	1.27	0	7
	PM_10_	2.20	1.41	0	9	2.13	1.37	0	7
	PM_2.5_	2.34	1.46	0	9	2.27	1.40	0	7
RB	NO_2_	1.43	0.96	0	5	1.39	0.95	0	4
	PM_10_	2.71	1.54	0	12	2.56	1.36	0	5
	PM_2.5_	3.03	1.75	0	15	3.09	1.66	0	6
UB	NO_2_	0.72	0.61	0	4	0.65	0.60	0	2
	PM_10_	1.08	0.77	0	5	1.07	0.76	0	3
	PM_2.5_	0.86	0.68	0	6	0.81	0.66	0	2
UT	NO_2_	0.67	0.61	0	4	0.58	0.57	0	2
	PM_10_	0.96	0.73	0	8	0.88	0.65	0	3
	PM_2.5_	1.25	0.84	0	8	1.23	0.81	0	3

Air quality only, using specific site types: Ind (Industrial: 11 sites), RB (rural background: 14 sites), UB (urban background: 48 sites), UT (urban traffic: 47 sites).

Table 3 shows that imputation consistently reduces the average number of rings required in the non-pollen measurements by a small amount. Also, as expected, due to the much lower number of pollen sites (15 pollen sites, see Table 1) and lack of imputation for such sites (see the Imputation of Missing Values section) the mean ring counts were significantly higher. The very high maximum ring counts (16) are due to early spring-time days (in all years) during which only a single pollen sensor in the UK had begun recording measurements after stopping for winter.

Table 4 shows ring counts for estimations performed on pollution data split into site types (see the Data Records section (Regional estimates subsection) for site type counts). As expected, more rings are required when using a smaller number of sites and the mean ring counts improve fractionally after imputation, with the maximum ring counts being significantly improved.

The performance of the estimation method at predicting what measurements would be in regions where sensors are missing has been tested. Each sensor measurement, at every timestamp (daily throughout 2016–2019), has been compared with an estimation made, for the region (in which the respective sensor is located) and timestamp, as if that particular sensor was missing. The list of resulting differences (sensor measurement minus estimated measurement) are described below.

Table 5 shows the summary statistics of the differences between original sensor values and estimated values for the selection of measurements described above. Table 6 shows the same summary statistics but for air quality values when split by sensor type. As raw values are used to calculate these differences, each measurement/row should be compared with its corresponding sensor value range displayed in Table 2. These results show that the mean differences are extremely low and that the greatest deviations from the mean are for the two pollen species. This is to be expected, as there is a low number of pollen sensors, gaps in the timeseries have not been filled using imputation, and pollen counts have a very complex response to environmental conditions. It should also be noted that the differences for NO_2_ have a large standard deviation (compared with the mean measurement value), indicating that there are environmental dependencies for this measurement too, which complicate the use of this technique. PM_10_ and PM_2.5_ do have the same issue, though it is less-pronounced for these pollutants. Stratifying the sensors according to site type (Table 6) indicates that a lot of this variance is driven by the urban traffic sites (which we would expect to have the most dependence on hyper-local conditions). Variance in estimated values for particulate pollutants does not show the same dependence on site type and, possibly, stratifying the sites may increase the variance for sites with lower numbers of sensors for these pollutants (such as for Industrial sites).

**Table 5:** Differences between region estimation and sensor values for daily means 2016–2019.

measur.	original				inc. imputed values			
	count	mean	std	skew	count	mean	std	skew
NO_2_	192534	−0.62	17.04	0.38	220611	−1.06	17.17	0.43
PM_10_	93189	−0.04	6.27	0.55	100809	−0.05	6.02	0.51
PM_2.5_	97477	−0.09	3.23	0.37	105192	−0.10	2.96	0.29
temp.	404324	0.01	1.25	−1.00	428073	0.02	1.19	−1.08
pressure	178919	−0.56	11.59	−0.54	185547	−0.42	11.55	−0.56
*Alnus* spp.	7386	−0.40	34.29	−1.92	—	—	—	—
Urticaceae	7387	−1.93	56.79	−0.63	—	—	—	—

**Table 6:** Differences between region estimation and sensor values for daily means 2016–2019.

type	measur	original				inc. imputed values			
		count	mean	skew	std	count	mean	skew	std
Ind	NO_2_	15671	−0.59	0	9.17	16071	−0.63	0	9.07
	PM_10_	9626	−0.04	1.03	9.05	10227	0.14	1.3	8.31
	PM_2.5_	8361	−0.31	−0.1	4.54	8766	−0.33	−0.6	4.33
RB	NO_2_	20655	0.6	0.76	6.43	21915	0.73	0.93	6.13
	PM_10_	5518	−1.41	−0.09	6.56	5844	−1.55	−0.14	6.23
	PM_2.5_	4672	−1.29	−0.76	5.81	4383	−1.71	−0.9	6.06
UB	NO_2_	70777	0.04	0.24	11.97	81816	−0.12	0.02	11.92
	PM_10_	35072	0.22	2.9	5.24	36525	0.23	2.31	5.03
	PM_2.5_	52451	−0.07	0.23	3.13	58440	−0.06	0.04	2.88
UT	NO_2_	79775	−0.23	0.23	18.9	94965	−0.18	0.26	17.81
	PM_10_	41061	−0.03	0.56	6.07	46752	0.11	0.23	5.71
	PM_2.5_	28700	0.06	0.09	4.18	30681	0.21	0.14	3.91

Air quality only, using specific site types: Ind (Industrial: 11 sites), RB (rural background: 14 sites), UB (urban background: 48 sites), UT (urban traffic: 47 sites).

## Usage Notes

Full instructions on how to use all code are included in the respective ‘readme.md’ files for each repository^18,19^. These include repository structure, requirements, usage, testing, how to contribute and licensing.

The AURN dataset is available via anonymous request through a web-based API. To access the MEDMI dataset users will need to register for an account on their server. This can be done by contacting them using the details on their webpage (https://www.data-mashup.org.uk/contact-us/).

## Data Availability

**Data download and processing scripts.** The scripts used to download, extract, and process the MEDMI and AURN datasets are implemented in Python and are available under the GPL-3.0 license^18^. Instructions on how to use them are included in the associated *README.md* files. **Regional estimations package.** The concentric regions algorithm described above is implemented in Python, using the publicly available GeoPandas library (http://geopandas.org/). We have made this regional estimations code available as a Python library, accessible under the MIT licence^19^. Instructions on how to use the package are included in the associated *README.md* file. This package receives inputs from users as three CSV files (site metadata, site measurement data and region metadata), and the algorithm iterates through each requested region and timestamp combination, outputting an estimation for each. As we present the concentric regions algorithm as a baseline method, this package is built in a modular fashion to facilitate users adding further regional estimation algorithms as new components, further to the two currently implemented (concentric regions and a simple shortest distance based algorithm). **EMEP modelling system.** The EMEP model source code is available to download from Zenodo^20^. The input files, and NAEI emissions used for this project are also available for download from Zenodo^21,22^.
